# Topological Advantage for Adsorbate Chemisorption on Conjugated Chains

**DOI:** 10.1021/acs.jpclett.5c03500

**Published:** 2026-01-27

**Authors:** Luis Martinez-Gomez, Raphael F. Ribeiro

**Affiliations:** Department of Chemistry and Cherry Emerson Center for Scientific Computation, 1371 Emory University, Atlanta, Georgia 30322, United States

## Abstract

Topological matter offers opportunities for control of charge and energy flow with implications for chemistry still incompletely understood. In this work, we study an ensemble of adsorbates with an empty frontier level (LUMO) coupled to the edges, domain walls (solitons), and bulk of a Su–Schrieffer–Heeger polyacetylene chain across its trivial insulator, metallic, and topological insulator phases. We find that two experimentally relevant observables, charge donation into the LUMO and the magnitude of adsorbate electronic friction, are significantly impacted by the electronic phase of the SSH chain and show clear signatures of the topological phase transition. Localized, symmetry-protected midgap states at edges and solitons strongly enhance electron donation relative to both the metallic and trivial phases, whereas, by contrast, the metal’s extended states, despite larger total DOS near the Fermi energy, hybridize more weakly with a molecular adsorbate near a particular site. Electronic friction is largest in the metal, strongly suppressed in gapped regions, and intermediate at topological edges, where hybridization splits the midgap resonance. These trends persist with disorder, highlighting their robustness, and suggest engineering domain walls and topological boundaries as pathways for employing topological matter in molecular catalysis and sensing.

## Graphical abstract

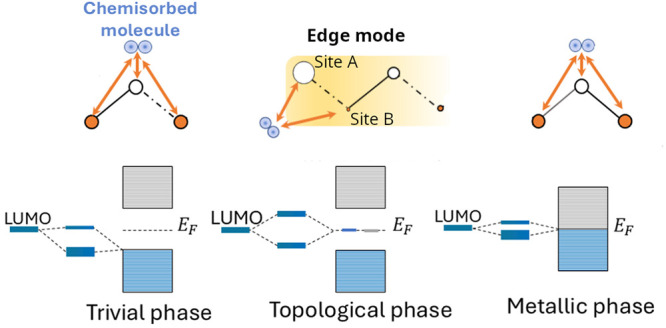

Topological insulators are electronic phases of matter with a bulk band gap and gapless surface states protected by global symmetries.^1–7^ These materials exhibit intriguing properties, including high surface carrier mobility^8–14^ with low power dissipation,^15,16^ and spin-polarized currents with unconventional textures.^17–20^ Potential applications include spintronics,^21–25^ quantum computing,^22,26,27^ and thermoelectrics.^28–32^ Recently, there has also been interest in utilizing topological insulators and semimetals in electrochemistry and chemical catalysis.^33–43^ Their highly localized conducting boundary modes may significantly influence surface reactions, and their properties are protected against local perturbations (e.g., lattice defects and impurities). For photocatalysis, high surface mobility is desirable because efficient charge separation and suppressed electron–hole recombination can enhance performance.^35,44–46^ Indeed, experimental^33,47–50^ and computational^39,51,52^ studies provide evidence that, under certain conditions, topological matter exhibits enhanced reactivity mediated by topologically protected boundary electrons. Nevertheless, the extent to which the surfaces of topological materials can be systematically exploited for high-efficiency, selective synthesis remains an open question.

In heterogeneous catalysis, adsorbed molecules undergo several processes on a solid surface.^53–56^ A central step is charge transfer between the extended material and the adsorbates.^57–59^ Orbital hybridization and electron donation into a molecular system influence adsorption,^53,60–62^ surface diffusion,^63,64^ and desorption, and can reduce energetic barriers for bond breaking or association.^65,66^ Likewise, adsorbate–surface energy exchange via - vibrational relaxation and excitation of boundary electron–hole pairs (EHPs)^67–78^ has been implicated in H_2_ relaxation on metal surfaces,^79,80^ CO_2_ reduction,^81–84^ and water oxidation.^85^ Here we focus on two mechanisms that are important to heterogeneous catalysis and can be directly analyzed in a minimal topological setting: (i) charge hybridization between molecular frontier orbitals and boundary electronic states and (ii) nonadiabatic vibrational energy dissipation (electronic friction) mediated by EHPs. We ask how these processes are modified when the substrate hosts symmetry-protected boundary modes versus trivial or metallic electronic structures.

We employ the Su–Schrieffer–Heeger (SSH) model^86,87^ as a minimal platform in which topological, metallic, and trivial insulating regimes arise within a single Hamiltonian, while still allowing controlled introduction of defects and systematic thermodynamic-limit analysis. Although the SSH chain is one-dimensional and we use a spinless, effective single-particle form, it faithfully captures the chiral (sublattice) symmetry and the associated boundary- and domain-wall-localized midgap orbitals that constitute the central ingredient of this work and that, as we show below, strongly modulate molecule–substrate interfacial properties.

This effective description is particularly well motivated for light-element π-conjugated polymers such as trans-polyacetylene: intrinsic spin–orbit coupling in the carbon *p*_*z*_ manifold is weak compared with eV-scale hoppings,^88^ and electron–electron interactions^89^ can be treated at an effective level through renormalized hopping amplitudes and onsite energies within a tight-binding picture. We briefly comment at the end on extensions to spinful models with strong spin–orbit coupling and strong correlations. The resulting minimal-model approach is therefore designed to isolate robust, design-relevant trends and in particular to clarify when and where enhanced hybridization and nonadiabatic dissipation should be expected, in a way that enables a direct interpretation of the effects reported here.

Within this controlled setting, we couple molecular adsorbates to SSH-class substrates to isolate and quantify how symmetry-protected midgap states (at finite edges and topological domain walls) govern molecule–substrate interfacial observables. Across metallic, trivial insulating, and topological regimes and in the presence of both trivial and topological defects, we compute adsorbate level broadening (hybridization), LUMO occupancy, and charge transfer for single and multiple adsorbates, thereby disentangling the roles of the - density of states and localized midgap modes. We further evaluate the electronic friction acting on an adsorbate within a linear response, via a kernel that captures electron–hole-pair-mediated vibrational damping at the contact. We show that localized midgap modes can (i) strongly enhance adsorbate hybridization and charge donation relative to both trivial insulating and metallic substrates and (ii) sharply increase vibrational energy dissipation through enhanced electronic friction upon crossing the topological transition; moreover, topological domain walls mitigate the potential measure-zero limitation of edge modes by providing a potentially extensive, chemically accessible set of such midgap states.

## Fano–Anderson SSH Model

The simplest scenario we considered consists of a molecule adsorbed in different regions of an open conjugated SSH chain (Figure 1). The isolated molecule has a closed shell and a single low-lying empty electronic orbital, while the SSH chain models the extended material.^86,90^ The molecule interacts with nearby lattice sites as in the Fano–Anderson model.^91,92^ To probe the roles of bulk versus boundary modes, we placed the molecule either near the chain edge or at the center (Figure 1).

**Figure 1:**
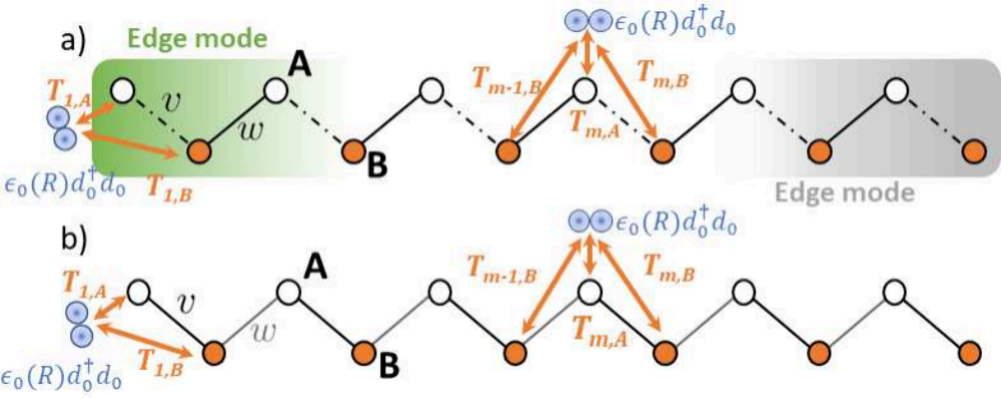
Schematic Fano–Anderson SSH setup. White and orange circles denote sublattices A and B, respectively, each with a single *p*_*z*_ orbital. Intra- and intercell hoppings (*v* and *w*) are tunable. A representative diatomic adsorbate couples either near an edge (*x*_*M*_ = *x*_*E*_) or at the chain center (*x*_*M*_ = *x*_*B*_). (a) Hybridization with edge/bulk modes in the topological phase (*v* < *w*). (b) Hybridization with a trivial insulator (*v* > *w*) or with the metallic limit (*v* = *w*), where states are delocalized.

The SSH model represents a polyacetylene chain by a 1D tight-binding lattice with two identical sites per unit cell, nearest-neighbor staggered hopping,^86^ and one electron per site. For a spinless open system, the isolated chain Hamiltonian is

1 $${Ĥ}_{SSH}=v\overset{N}{\underset{j=1}{\sum }}\left({\mathrm{d̂}}_{j,B}^{†}{\mathrm{d̂}}_{j,A}+{\mathrm{d̂}}_{j,A}^{†}{\mathrm{d̂}}_{j,B}\right)+w\overset{N-1}{\underset{j=1}{\sum }}\left({\mathrm{d̂}}_{j+1,A}^{†}{\mathrm{d̂}}_{j,B}+{\mathrm{d̂}}_{j,B}^{†}{\mathrm{d̂}}_{j+1,A}\right)$$

where *v* (*w*) is the intracell (intercell) hopping, *N* is the number of unit cells, and ${\mathrm{d̂}}_{j,\alpha }^{†}$ $\left({\mathrm{d̂}}_{j,\alpha }\right)$ creates (annihilates) an electron on sublattice α ∈ {*A*, *B*} in cell *j*. The gapped phases correspond to *v* ≠ *w*: *v* > *w* is a trivial insulator without boundary-localized modes, whereas *v* < *w* yields a gapped system with two zero-energy boundary modes.^4,86^

In a fully spinful description of a dimerized π-conjugated chain (e.g., polyacetylene), each spatially localized midgap bound state corresponds to a localized *π* frontier orbital. Depending on filling and interactions, this orbital can be singly occupied and carry an unpaired electron, i.e., it acts as a radical center in the language of conventional organic chemistry.^93^ In the present work, we use a spinless SSH Hamiltonian, which can be viewed as isolating a single spin channel. In a more realistic spinful model, the same spatial orbital would be 2-fold spin-degenerate. Hence, the topological midgap states of the SSH chain are tight-binding analogues of chemically familiar radical-like frontier orbitals, with the dimerization ratio (*w* > *v* vs *w* < *v*) controlling whether such orbitals are present or absent. In what follows, we focus on how the existence, localization, and filling of these midgap frontier orbitals modulate charge transfer and electronic friction between the conjugated backbone and the adsorbate.

The isolated effective single-level adsorbate is described by ${Ĥ}_{0}\left(R\right)=\varepsilon_{0}\left(R\right){\mathrm{d̂}}_{0}^{†}{\mathrm{d̂}}_{0}$ , where ${\mathrm{d̂}}_{0}$ annihilates an electron in the adsorbate (LUMO) and *R* is an internal vibrational coordinate. The full Hamiltonian for a single species adsorbed to the SSH chain is

2 $$Ĥ\left(R,x_{M}\right)={Ĥ}_{SSH}+{Ĥ}_{0}\left(R\right)+Ŵ\left(x_{M}\right)$$

where *x*_*M*_ ∈ {*x*_*B*_, *x*_*E*_} labels bulk- or edge-coupled configurations. The interaction Hamiltonian $Ŵ\left(x_{M}\right)$ describes hopping between the chain and the adsorbate orbital:

3 $$Ŵ\left(x_{E}\right)=T_{1,A}{\mathrm{d̂}}_{1,A}^{†}{\mathrm{d̂}}_{0}+T_{1,B}{\mathrm{d̂}}_{1,B}^{†}{\mathrm{d̂}}_{0}+\mathrm{h.c.}$$

4 $$Ŵ\left(x_{B}\right)=T_{N/2,A}{\mathrm{d̂}}_{N/2,A}^{†}{\mathrm{d̂}}_{0}+T_{N/2,B}{\mathrm{d̂}}_{N/2,B}^{†}{\mathrm{d̂}}_{0}+T_{N/2-1,B}{\mathrm{d̂}}_{N/2-1,B}^{†}{\mathrm{d̂}}_{0}+\mathrm{h.c.}$$

where *N* is taken even for simplicity and the couplings $T_{s,\alpha }\in R$ are positive and much smaller than *v* and *w*. Figure 1 summarizes the considered configurations.

Polyacetylene chains may host both trivial and topological lattice defects.^86,87^ The latter are domain walls separating trivial (*v* > *w*) and topological (*v* < *w*) regions and support midgap localized modes. We therefore also consider a scenario in which multiple molecules interact with such defects. In the considered static limit (immobile defects), *N*_*d*_ solitons centered at sites $m_{1},...,m_{N_{d}}$ are modeled by site-dependent nearest-neighbor hoppings

5 $$t_{n}\equiv H_{SSH}^{n,n+1}=\frac{w+v}{2}+\frac{v-w}{2}\overset{N_{d}}{\underset{i=1}{\sum }}\phi_{n}^{\left(m_{i}\right)}$$

with^94^

6 $$\phi_{n}^{\left(m_{i}\right)}={\left(-1\right)}^{n}\;\tanh\left(\frac{n-m_{i}}{\xi }\right)$$

where ξ is the soliton width. Although solitons in real polyacetylene generally arise as thermally activated defects, here, we treat them as fixed static inhomogeneities and work at half filling with a fixed electron number. This approximation allows us to examine the fundamental role of topological defects without introducing additional assumptions regarding thermally driven soliton formation and dynamics.

In disordered samples, each adsorbate is placed at a soliton center *m*_*i*_ and couples to the nearest site with strength $T_{m_{i}}$ and to its two nearest neighbors *m*_*i*_ ± 1 with $T_{m_{i}}/3$ , so each adsorbate interacts with three sites. The adsorbates are typically sufficiently far from each other that the adsorbate ensemble Hamiltonian is the straightforward generalization of ${Ĥ}_{0}$ to multiple noninteracting adsorbates.

## Observables

To quantify charge transfer and hybridization for a single adsorbate, we computed the adsorbate population *n*_0_(*R*, *x*_*M*_) for a half-filled SSH chain at zero temperature with the Fermi energy set to μ = 0. Defining the occupied single-particle projector as ${\mathrm{P̂}}_{occ}\left(R,x_{M}\right)=\Theta \left(\mu -Ĥ\left(R,x_{M}\right)\right)$ where Θ is the Heaviside step function, we write

7 $$n_{0}\left(R,x_{M}\right)=\left\langle \varepsilon_{0}\right|{\mathrm{P̂}}_{occ}\left(R,x_{M}\right)\left|\varepsilon_{0}\right\rangle =\underset{\lambda :{\text{ϵ}}_{\lambda }\left(R,x_{M}\right)<\mu }{\sum }{\left|\left\langle \varepsilon_{0}\right|\lambda \left(R,x_{M}\right)\rangle \right|}^{2}$$

where |*ε*_0_⟩ is the single-particle orbital associated with the adsorbate creation operator ${\mathrm{d̂}}_{0}^{†}$ , and {|λ­(*R*, *x*_*M*_)⟩} are eigenstates of $Ĥ\left(R,x_{M}\right)$ with energies {ϵ_λ_(*R*, *x*_*M*_)}. In practice, we take μ → 0^–^ to resolve any zero-mode degeneracies in the topological phase. We obtain *n*_0_(*R*, *x*_*M*_) by numerical diagonalization and, where appropriate, perturbation theory.

For multiple adsorbates and a variable number of defects, we assess chemisorption via the mean LUMO occupancy

$$\left\langle n_{0}\right\rangle =\frac{1}{N_{ad}}\overset{N_{ad}}{\underset{i=1}{\sum }}n_{0i}$$ 8

where *n*_0*i*_ is the *i*th adsorbate LUMO population.

To probe vibrational energy exchange with the substrate, we consider the adsorbate vibrational degree of freedom *R* as a dynamical variable, modeled as a harmonic oscillator with mass *m*, frequency ω, and displacement from equilibrium (at the electronic ground-state) *R*. We assume a linear vibronic coupling of the adsorbate level^75,79^

9 $${Ĥ}_{0}\left(R\right)=\varepsilon_{0}\left(R\right){\mathrm{d̂}}_{0}^{†}{\mathrm{d̂}}_{0}+U_{0}\left(R\right)$$

10 $$\varepsilon_{0}\left(R\right)=\varepsilon_{d}+gR,,U_{0}\left(R\right)=\frac{1}{2}m\omega^{2}R^{2}$$

where *ε*_*d*_ is the LUMO energy at *R* = 0, *g* = *∂ε*_0_/*∂R* is the (Condon) linear vibronic coupling, and the dependence on *x*_*M*_ is omitted for notational simplicity.

In contact with an extended electronic system, vibrational relaxation can proceed via nonadiabatic excitation of electron–hole pairs (EHPs).^69,75^ Under the standard separation of time scales where the electronic subsystem equilibrates much faster than the nuclear motion,^95,96^ the vibrational energy loss rate is captured by an electronic kernel.^68,69,72,75,97–99^ For a single coordinate *R*,

11 $$\gamma \left(R\right)=-\pi \hbar \int_{-\infty }^{\infty }d\text{ϵ}\Xi \left(\text{ϵ};R\right)\frac{\partial f_{T}\left(\text{ϵ}\right)}{\partial \text{ϵ}}$$

12 $$\Xi \left(\text{ϵ};R\right)=Tr\left[\frac{\partial Ĥ\left(R\right)}{\partial R}\mathrm{P̂}\left(\text{ϵ};R\right)\frac{\partial Ĥ\left(R\right)}{\partial R}\mathrm{P̂}\left(\text{ϵ};R\right)\right]$$

where the trace is over the single-particle electronic space and $\mathrm{P̂}\left(\text{ϵ};R\right)=\underset{\lambda }{\sum }\delta (\text{ϵ}-{\text{ϵ}}_{\lambda }\left(R\right))\left|\lambda \left(R\right)\right\rangle \left\langle \lambda \left(R\right)\right|$ is the spectral projector. For the Hamiltonian in ([Disp-formula eq2]) with ([Disp-formula eq9]), assuming (i) only the adsorbate level *ε*_0_(*R*) depends on *R* (no *R*-dependence in the couplings *T*_*s*,α_, i.e., with non-Condon effects neglected) and (ii) linear response about a fixed *R*, the friction reduces to

13 $$\gamma \left(R\right)=-\pi \hbar g^{2}\int_{-\infty }^{\infty }d\text{ϵ}\underset{\lambda ,\lambda^{\prime }}{\sum }{\left|\left\langle \lambda \left(R\right)\right|\varepsilon_{0}\rangle \right|}^{2}\delta [\text{ϵ}-{\text{ϵ}}_{\lambda }\left(R\right)]\times {\left|\left\langle \lambda^{\prime }\left(R\right)\right|\varepsilon_{0}\rangle \right|}^{2}\delta [\text{ϵ}-{\text{ϵ}}_{\lambda^{\prime }}\left(R\right)]\frac{\partial f_{T}\left(\text{ϵ}\right)}{\partial \text{ϵ}}$$

Equation 13 corresponds to the Head–Gordon–Tully electronic-friction tensor, i.e., the *equilibrium, Markov* isolated system limit for nuclear motion coupled to a dense manifold of electronic excitations.^72^ We note that more general nonequilibrium open-system treatments can yield non-Markovian friction and additional force contributions beyond a simple Markovian friction tensor. Nonetheless, in the assumed equilibrium, isolated system limit with fast decoherence (induced by other molecular or substrate degrees of freedom), the HGT expression has been shown to be consistent with more general open-system formulations of electronic friction.^300,301^

In our simulations, the electronic environment is represented by a finite SSH chain, so the electronic spectrum is discrete at finite *N*. To obtain a continuous spectrum, and hence a well-defined Markovian friction, we broaden each discrete level by a Gaussian of width σ following standard practice,^70,72^ replacing the Dirac delta functions by normalized Gaussians

14 $$\delta (\text{ϵ}-{\text{ϵ}}_{\lambda })\to \frac{1}{\sigma \sqrt{2\pi }}\;\exp\left[-\frac{{(\text{ϵ}-{\text{ϵ}}_{\lambda })}^{2}}{2\sigma^{2}}\right]$$

thereby yielding a smooth LDOS *P*(ϵ, *R*) and numerically stable friction coefficients.^100^

In this work, we use an *N*-independent broadening parameter chosen on the order of the thermal smearing scale, σ ∼ *k*_*B*_ *T*.^101^ Importantly, our conclusions do not depend on this choice: the friction coefficients are robust with respect to moderate changes in σ and with respect to increasing the chain size *N* (see the Supporting Information).

## Bulk and Boundary Chemisorption

In what follows, we examine the chemisorption of a single adsorbate on the SSH chain under various conditions.

To place our parameter choices in the context of realistic 1D conjugated systems, we note that tight-binding descriptions of polyacetylene and polyacetylene-like chains^102–105^ are broadly consistent with nearest-neighbor π-electron hopping scales of a few eV and a moderate bond-alternation (dimerization) contrast. Representative values such as *v* = 2.5 eV and *w* = 3.5 eV^102^ imply a hopping ratio *w*/*v* of order unity with appreciable bond alternation, consistent with the scale of parameters used in the present model.

Because our central conclusions are governed by dimensionless ratios such as the dimerization ratio

$$r\equiv \frac{w}{v}$$

the dimensionless LUMO energy *ε*_0_/*v*, and hopping *T*/*v*, the predicted midgap-state-enabled enhancement of hybridization and electronic friction is robust across the range of *r* explored here. We therefore expect the reported trends to be transferable.

Our analysis covers the trivial (*r* < 1), metallic (*r* ≃ 1), and topological (*r* > 1) regimes. We emphasize our primary objective is not to model a specific material parameter set but to map how the topological phase transition and the appearance of boundary-localized midgap states modulate charge hybridization and electronic friction for an adsorbate across a broad, physically motivated range of *r*.

Throughout this section, the molecular geometry is fixed (*R* = 0). The adsorbate is therefore characterized by its low-lying level energy *ε*_0_ and placement *x*_*M*_. We also note that all equilibrium observables reported below are converged with respect to the SSH chain length: increasing the number of unit cells *N* leaves the electron occupancy essentially unchanged so that the finite chain adopted here represents the system in the thermodynamic limit (see the Supporting Information for verification).

Figure 2a,b reports the LUMO occupation *n*_0_(*R*, *x*_*M*_) at 0 K and half filling (μ = 0^–^) for adsorption near the edge (*x*_*M*_ = *x*_*E*_) or at the bulk center (*x*_*M*_ = *x*_*B*_). Figure 2a shows that the trivial insulator exhibits negligible electron sharing at the edge, whereas the topological phase displays strong hybridization with a sharp rise of *n*_0_(*R*, *x*_*E*_) upon entering *r* > 1. The enhancement is most pronounced when *ε*_0_ is nearly resonant with the boundary mode. Conversely, Figure 2b shows that for adsorption in the metallic bulk significant *n*_0_(*R*, *x*_*B*_) occurs only in a narrow neighborhood of the topological transition critical point (*r* ≈ 1), where the density of occupied states near *ε*_0_ is largest. While the metallic phase maximizes bulk adsorption, boundary chemisorption in the topological phase remains the optimal overall scenario for hybridization under the considered conditions as max *n*_0_(*R*, *x*_*B*_) < max *n*_0_(*R*, *x*_*E*_) = 0.5. Notably, appreciable adsorbate-edge hybridization persists even when *ε*_0_ ≫ 0.

**Figure 2:**
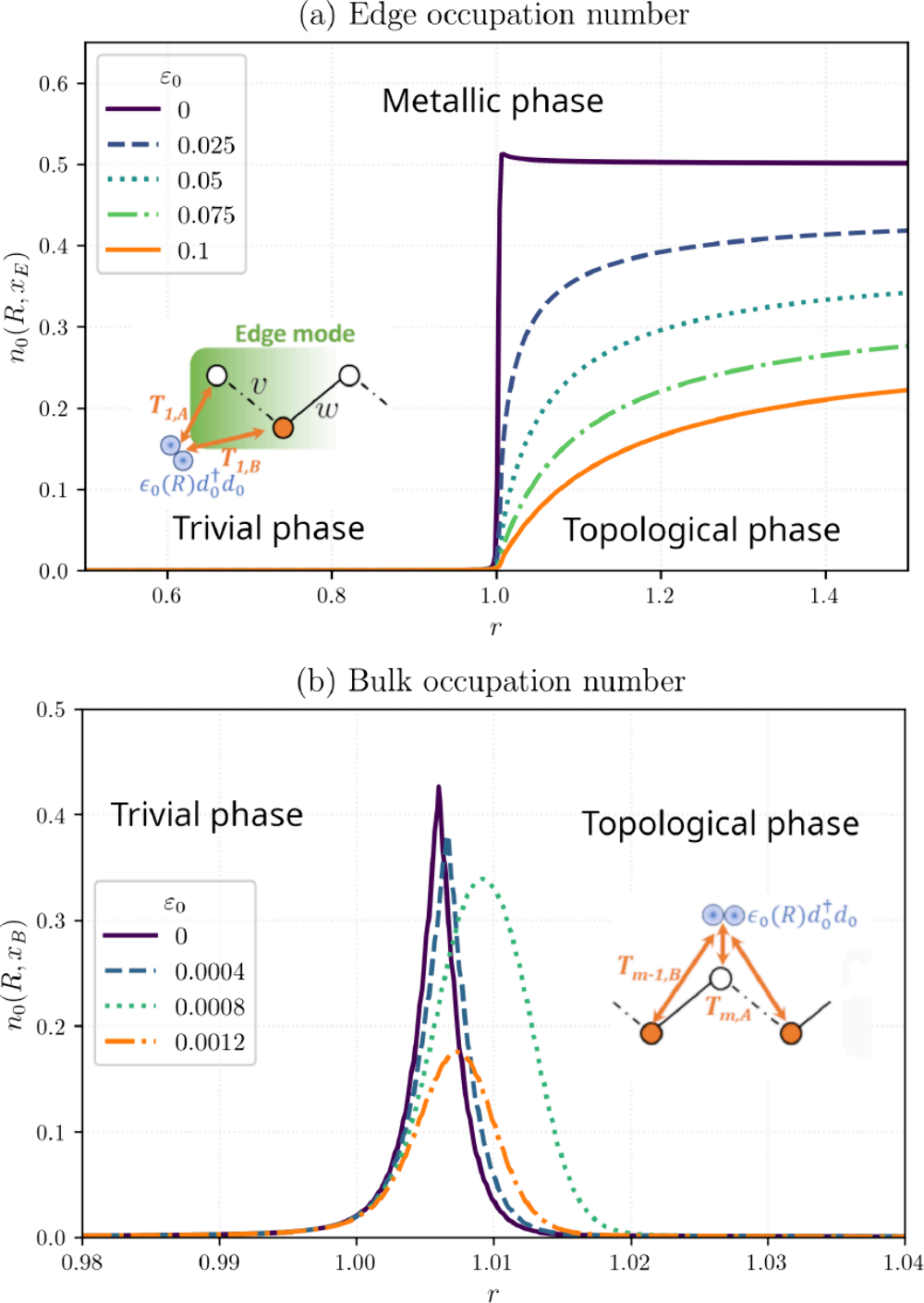
LUMO occupation near the (a) edge (*T*_1,*A*_ = *T*_1,*B*_/3 = 0.1) and (b) bulk center (*T*_*N*/2,*A*_ = *T*_*N*/2+1,*B*_/3 = *T*_*N*/2–1,*B*_/3 = 0.1) of the chain for trivial (*r* < 1), metallic (*r* ≈ 1), and topological (*r* > 1) phases. Parameters: *N* = 800, *v* = 10, μ = 0.

The robustness of the topological enhancement is highlighted in Figure 3, which directly compares edge adsorption in the topological phase to bulk adsorption in the metallic phase over varying *ε*_0_ and couplings *T*_*m*,*A*_. Across all cases examined, hybridization with metallic bulk states yields a lower *n*_0_ than coupling to the topological edge mode, despite the larger metallic DOS near *ε*_0_. Notably, edge *n*_0_(*R*, *x*_*E*_) remains substantial even when *T*_1,*A*_ ≪ *v*, *w*.

**Figure 3:**
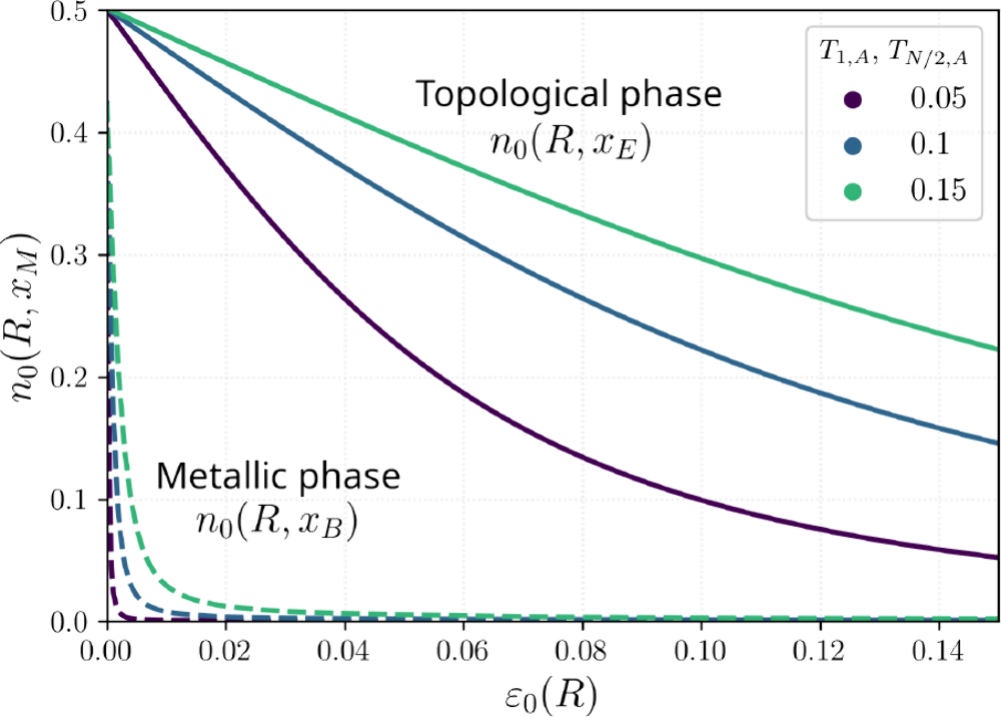
LUMO occupation vs adsorbate level *ε*_0_: topological edge (*w* = 15, solid lines) compared with metallic bulk (colors denote *r* = 1.00814 [purple], 1.00597 [blue], 1.00465 [blue-green]) for several *T*_*m*,*A*_. Parameters: *N* = 800, *v* = 10, μ = 0.

## Discussion

In the topological phase and near resonance (*ε*_0_ ≈ 0), the adsorbate couples predominantly to the nearest edge mode. For *T*_1,*A*_, *T*_1,*B*_ ≪ *v*, *w* and *w*/*v* – 1 ≫ 0, the hybridized edge–LUMO doublet is well isolated from bulk continua, yielding the symmetric occupancy *n*_0_(*R*, *x*_*E*_) ≈ 0.5. In the metallic regime (*r* ≃ 1), *ε*_0_ ≈ 0 is near-degenerate with many occupied/unoccupied extended states and the resulting Fano line shape^91^ in the local molecular DOS produces a lower maximal *n*_0_ ≈ 0.4. Off resonance, bulk adsorption admits the standard second-order estimate

15 $$n_{0}\left(R,x_{B}\right)\approx \underset{E_{\kappa }^{\left(0\right)}<\mu }{\sum }\frac{{\left|T_{c}\cdot u_{\kappa ,c}\right|}^{2}}{{\left(\varepsilon_{0}-E_{\kappa }^{\left(0\right)}\right)}^{2}}$$

where $E_{\kappa }^{\left(0\right)}$ denote bare SSH eigenenergies, *c* = *N*/2, ***T***_*c*_ and ***u***_κ,*c*_ are

$$T_{c}\equiv \left(\begin{array}{l} T_{c,A}\\ T_{c,B}\\ T_{c-1,B} \end{array}\right),,u_{\kappa ,c}\equiv \left(\begin{array}{l} a_{c,\kappa }\\ b_{c,\kappa }\\ b_{c-1,\kappa } \end{array}\right)$$

and *a*_*j*,κ_ and *b*_*j*,κ_ are the amplitudes of eigenstate κ on sublattices *A*/*B* at cell *j*. The sum runs over occupied states at *T* = 0 K. Away from criticality the occupied bulk states with non-negligible weight at the molecule are delocalized and detuned from *ε*_0_, suppressing *n*_0_(*R*, *x*_*B*_). Conversely, when *r* ≈ 1, the increased density of states and small denominators in ([Disp-formula eq15]) enhance *n*_0_.

In summary (Figure 4), we find that strong spatial localization of the topological midgap modes enables robust hybridization and charge donation to an edge-bound adsorbate. By contrast, despite its larger DOS, the metallic phase shows weaker hybridization and charge transfer into the adsorbate LUMO at a given local contact because extended states dilute the local overlap between the adsorbate low-lying orbital and the SSH chain.

**Figure 4:**
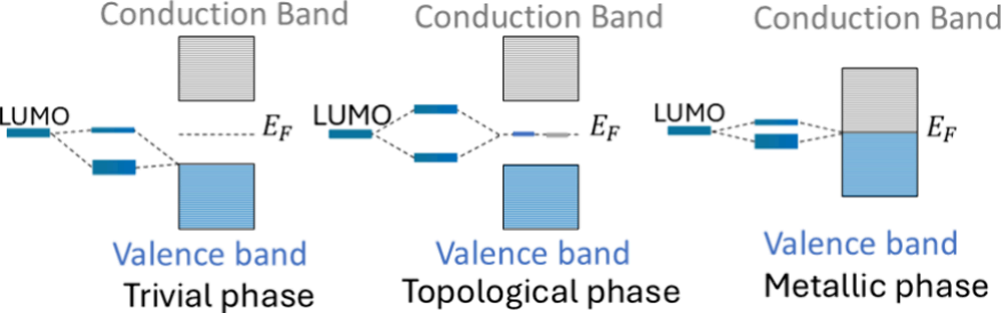
Schematic hybridization between the adsorbate LUMO and SSH states in the trivial (left), topological (center), and metallic (right) regimes.

## Chemisorption on Solitons and Trivial Defects

In an ideal SSH chain, midgap (*E* ≈ 0) modes occur only at the edges of the topological phase *r* > 1. At finite temperature, bulk topological defects known as solitons (domain walls)^86,87,94^ arise and bind additional exponentially localized midgap states in the interior. These localized states may also hybridize efficiently with nearby molecular orbitals, enabling the donation of charge to an adsorbate. In contrast, a metallic SSH chain (*r* = 1) lacks localized midgap modes but has greater density of states of near-resonant levels with the adsorbate, and in the presence of trivial defects (e.g., impurities) may also have local resonances that could hybridize effectively with adsorbates. We therefore compare multiadsorbate chemisorption on the following substrates: (i) a topological chain with multiple solitons (soliton ensemble), (ii) an ordered metallic chain, and (iii) a disordered metallic chain with trivial defects.

We report the average LUMO occupancy ⟨*n*_0_⟩ (eq 8) for *N*_ad_ adsorbates on a chain of *N* unit cells at 0 K and half filling. Domain walls have width ξ ∈ {5, 7, 10} (corresponding to the localization length of their midgap states, see eq 6). In the soliton ensemble simulations reported in Figure 5a, each adsorbate sits at a soliton center, and *N*_ad_ equals the number of domain walls *N*_*DW*_ (including the two edges). In the metallic phase with trivial defects, we set *r* = 1 and introduce a dilute ensemble of defects by drawing small near-zero on-site potentials from a Gaussian distribution, applied to a random subset of sites to mimic weak nontopological disorder. Adsorbates are then placed at defect locations drawn from a uniform distribution over bulk sites. Finally, in the zero-disorder metal scenario, *r* = 1 and the adsorbates are placed at uniformly random bulk sites. Unless otherwise specified, these simulations employed *N* = 3000, *v* = 10, *T*_*m*_ = 0.1, and averages over 25 realizations with fixed electron number.

**Figure 5:**
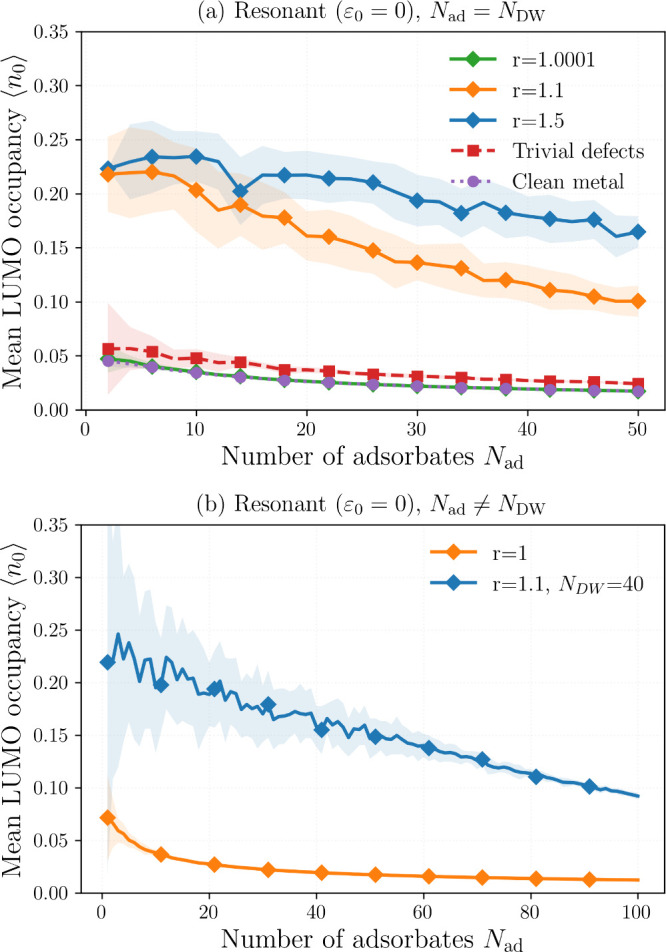
Resonant case *ε*_0_ = 0. (a) $\left\langle n_{0}\right\rangle$ vs number of adsorbates (defects) for soliton ensembles at three dimerizations (*r* = 1.5, 1.1, 1.0001), compared to (i) metal (*r* = 1 with the same number of on-site defects) and (ii) disorder-free metal (*r* = 1, no defects). Shaded regions represent fluctuations over realizations of the disordered systems, and we have set the number of adsorbates to be equal to the number of domain walls *N*_*ad*_ = *N*_*DW*_. (b) $\left\langle n_{0}\right\rangle$ vs *N*_ad_ for a fixed set of 40 domain walls (topological) or none (zero-disorder metal), and adsorbates placed at random sites. Parameters: ξ = 7, *N* = 3000, *v* = 10, *T*_*m*_ = 0.1, and 25 realizations.

Across all conditions, chains with domain walls yield systematically larger $\left\langle n_{0}\right\rangle$ than metallic chains with trivial defects (Figure 5a). The enhancement tracks soliton localization: larger *r* (stronger dimerization) narrows midgap wave functions and increases adsorbate–chain overlap.^86^ Near criticality (*r* = 1.0001) midgap states broaden, and charge transfer is reduced. Fluctuations reflect variability from soliton–antisoliton spacing and boundary proximity.

The near plateau $\left\langle n_{0}\right\rangle \approx 0.25$ at *r* = 1.1 (Figure 5a) can be explained as follows. At half filling and with particle–hole symmetry, each isolated domain wall binds one midgap state at zero energy which is half filled on average. Given that any adsorbate positioned in the neighborhood of a particular soliton can reach at most *n*_0_ ≈ 0.5 when *ε*_0_ ≥ 0 (Figure 3), averaging over all adsorbates gives a low-coverage plateau of ⟨*n*_0_⟩ ≈ (0.5) × (0.5) = 0.25. As the number of domain walls increases, domain–wall interactions lead to the observed decay in ⟨*n*_0_⟩. Metals (*r* = 1) with trivial defects behave similarly to clean metals, i.e., both give much lower $\left\langle n_{0}\right\rangle$ relative to the system with topological defects, thus indicating that nontopological near-zero resonances do not generically replicate midgap-assisted donation and instead hybridize significantly with metal extended states rather than to the adsorbate orbital.

Figure 5b fixes 40 solitons but randomizes adsorbate positions; hence, the adsorbates may or may not land on a domain wall. At low coverage the same plateau ⟨*n*_0_⟩ ≈ 0.25 emerges but with large variance: realizations in which adsorbates overlap midgap states yield *n*_0_ ∼ 0.5, whereas those on bulk sites yield *n*_0_ ∼ 0. This two-population mixture (soliton-bound vs bulk-bound) produces both the 0.25 mean and the broad fluctuations (shaded blue region). In contrast, ordered metallic chains give substantially lower mean LUMO occupancy at all coverages, underscoring the chemisorption advantage conferred by topological midgap states.

Off resonance (*ε*_0_ > 0; Figure 6) the topological advantage persists but is reduced. The dependence on ξ is weak-to-moderate: narrower domain walls (smaller ξ) produce more localized midgap states and slightly larger ⟨*n*_0_⟩ due to enhanced wave function overlap. In contrast, the trivial-defect metal behaves similarly to the ordered metal, indicating that nontopological near-zero local resonances do not generically replicate the midgap-assisted donation of domain walls.

**Figure 6:**
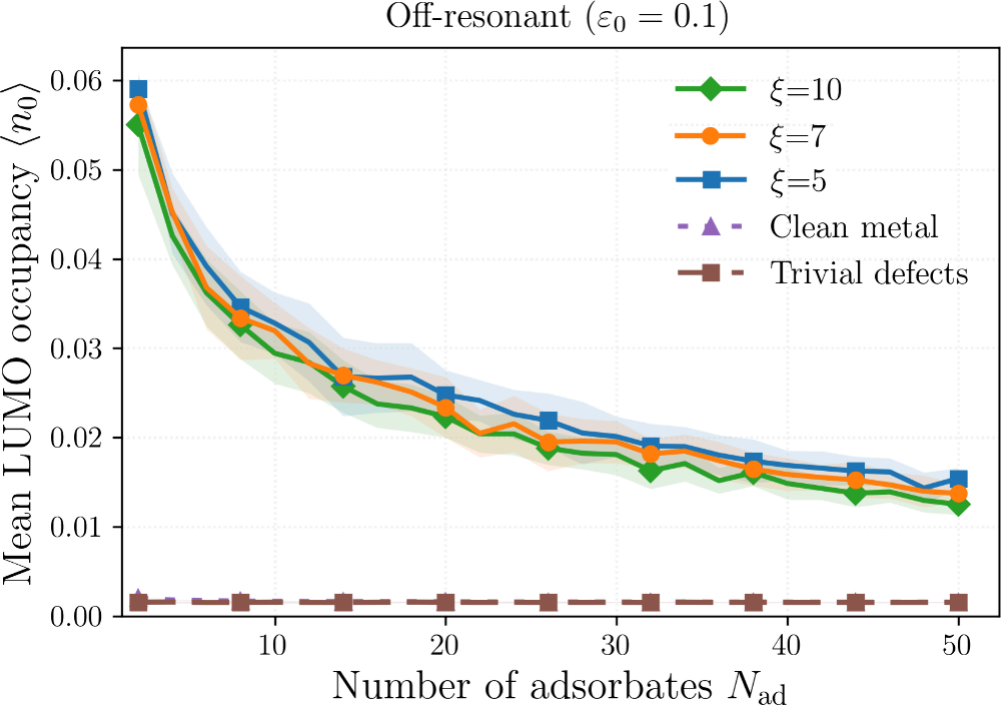
Off-resonant case *ε*_0_ = 0.1: $\left\langle n_{0}\right\rangle$ vs *N*_ad_ and defect content. Parameters: *N* = 3000, *v* = 10, *T*_*m*_ = 0.1.

## Topological Phase Transition Signature on Adsorbate Electronic Friction

Using the adiabatic orbitals from diagonalizing eq 2 with $\varepsilon_{0}\left(R\right)={\text{ϵ}}_{d}+Rg\sqrt{m\omega /\hbar }$ , we construct the local molecular density of states *P*(ϵ, *R*) and evaluate the electronic friction γ­(*R*) via eq 13 for the metallic (*r* = 1), topological (*r* > 1), and trivial (*r* < 1) phases at temperature 1/β = 0.015. As explained above, *P*(ϵ, *R*) is obtained by Gaussian broadening of each adiabatic level ϵ_λ_(*R*) with width σ = 0.0225. While σ is not uniquely defined,^106^ this choice yields smooth nonoscillatory *P*(ϵ, *R*) and γ­(*R*) sufficient for qualitative trends across phases (see the Supporting Information for a sensitivity analysis). For a dilute impurity concentration and fixed electron number in the polyacetylene chain, we treat the system effectively as intrinsic.^107^ We modeled the interacting adsorbate as a hole-like impurity and set the Fermi level μ in the HOMO–LUMO midgap at half filling (spinless electrons, for *N* unit cells). Because hybridization shifts the spectrum, μ is tracked self-consistently as a function of *R*; see Figure 8b.

Figure 7a shows γ­(*R*) vs *ε*_0_(*R*) for representative placements and dimerization *r* = *v*/*w*. The metallic phase exhibits the largest friction, consistent with a high LDOS at μ and abundant low-energy electron–hole excitations. In the topological phase at the edge, the localized boundary mode enhances friction relative to a gapped bulk but remains below the metallic value. The reduction arises from the hybridization-induced splitting between the edge mode and the LUMO: a two-level avoided crossing produces a PDOS doublet ⟨*ε*_0_|*P*(ϵ, *R*)|*ε*_0_⟩ separated by $\Delta E∼2O\left(T_{1,A}\right)$ , which depresses the LDOS at μ and suppresses electron–hole pair generation (Figure 8a) near the Fermi level.

**Figure 7:**
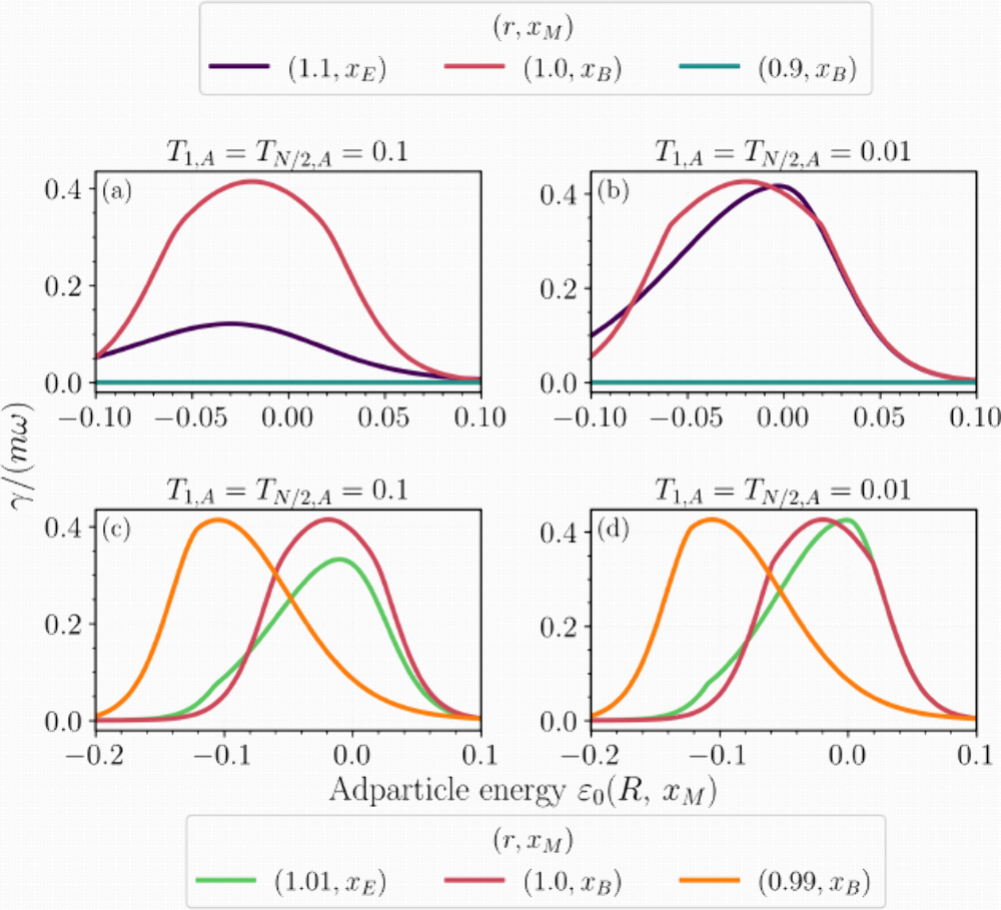
Electronic friction γ­(*R*) (eq 13) across phases. (a) Topological edge (*x*_*E*_; purple, *r* = 1.1), metallic bulk (*x*_*B*_; magenta, *r* = 1), and trivial bulk (*x*_*B*_; teal, *r* = 0.9) at *T*_1,*A*_ = *T*_*N*/2,*A*_ = 0.1. (b) Same as (a) with weaker coupling *T*_1,*A*_ = *T*_*N*/2,*A*_ = 0.01. (c, d) Near criticality: topological edge (*x*_*E*_; green, *r* = 1.01) and trivial bulk (*x*_*B*_; orange, *r* = 0.99) vs *ε*_0_(*R*) at *T*_1,*A*_ = 0.1 (c) and *T*_1,*A*_ = 0.01 (d). Parameters: *v* = 10, σ = 0.0225, *ε*_*d*_ = 0.15, *g* = 0.02, β = 1/0.015, *N* = 800, with *T*_*N*/2–1,*B*_ = *T*_*N*/2,*B*_ = *T*_*N*/2,*A*_/3.

**Figure 8:**
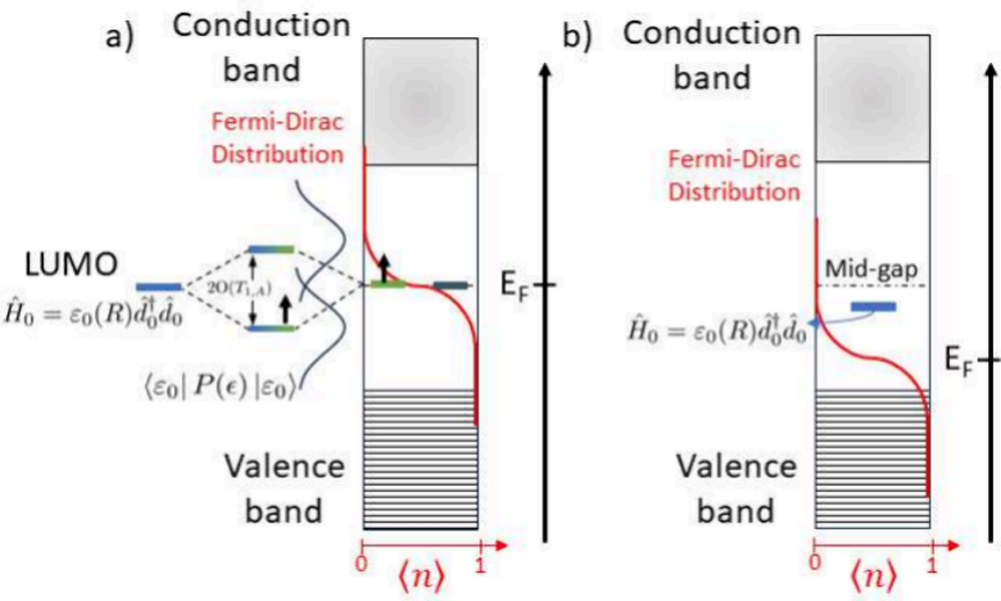
(a) Topological edge: hybridization between the localized edge state and the adsorbate level produces a PDOS (blue solid lines) doublet ⟨*ε*_0_|*P*(ϵ, *R*)|*ε*_0_⟩ split by $∼2O\left(T_{1,A}\right)$ . Coupling to the distant edge is negligible. (b) Trivial insulating bulk: with half filling, *E*_*F*_ sits near midgap; the adsorbate level is far from the band edges, yielding vanishing γ­(*R*).

At smaller adsorbate–edge coupling *T*_1,*A*_ = 0.01 (Figure 7b), the splitting Δ*E* diminishes, partially restoring LDOS at *E*_*F*_ and increasing γ­(*R*) relative to the stronger-coupling case. Note this enhancement occurs as one reduces a strong coupling. For sufficiently weak coupling, γ ultimately decreases with *T*_1,*A*_ as the adsorbate decouples (see the Supporting Information for further discussion).

For a sizable SSH gap |*w* – *v*| = 1 (e.g., *v* = 10, *w* = 9), thermal carriers are exponentially suppressed and the adsorbate level lies far from the valence–conduction continua (Figure 8b). With μ pinned near the midgap, γ­(*R*) is negligible in the trivial phase, consistent with the absence of low-energy electron–hole channels. Likewise, in the topological phase but *in the bulk* (away from edges), γ­(*R*) is similarly suppressed; we therefore omit those curves from Figure 7 for clarity. In such gapped settings, vibrational energy relaxation is likely dominated by phonon-mediated channels in the insulating substrate.^108,109^

Figures 7c,d examines γ­(*R*) as *ε*_0_(*R*) sweeps across band edges close to criticality. In the topological phase, the friction grows when *ε*_0_(*R*) approaches the band edges because both conduction and valence states contribute to *P*(ϵ, *R*) near *E*_*F*_. In the trivial phase, the friction peak shifts toward the valence band edge. The maximum occurs when the adsorbate aligns with the bulk HOMO, *ε*_0_(*R*) ≈ *w* – *v*. For *v* = 10 and *r* = 0.99 (i.e., *w* = 9.9), this gives *ε*_0_(*R*) ≈ −0.1. Reduced *T*_1,*A*_ narrows the avoided crossing even near criticality, mirroring the trend in Figure 7b.

We studied chemisorption of adsorbate species with an empty frontier level coupled to a polyacetylene SSH chain across its trivial (*r* < 1), metallic (*r* = 1), and topological (*r* > 1) regimes. We extensively analyzed (i) electron donation into the adsorbate LUMO and (ii) nonadiabatic electronic friction experienced by an adsorbate vibrational coordinate as a function of the adsorbate position along the chain and its internal geometry. We further contrasted topological domain walls (solitons) with nontopological, trivial defects in the metallic phase.

We find a robust advantage for electron donation in the topological phase relative to those of both the metallic and trivial phases. This is explained by the fact that exponentially localized midgap states at edges (and at solitons) have large wave function amplitude at the adsorption site, which enhances adsorbate–substrate hybridization at resonance. By contrast, although the metallic phase has a larger total density of states near the Fermi level, its extended bulk states are spatially dilute at any one site, weakening local hybridization and reducing charge transfer. Importantly, merely generating localized states is *necessary but not sufficient*: the donation depends also on resonance alignment, spatial overlap, and occupancy. Topological midgap modes satisfy these criteria in a robust, symmetry-protected manner, whereas trivial near-zero resonances in a metal generally do not. Consistent with this picture, solitons in the topological phase substantially outperform trivial defects in a metal. Near criticality (weak dimerization regime *r* ≈ 1) the midgap states broaden, and the advantage correspondingly diminishes.

Adsorbate electronic friction provides a complementary dynamic signature of the electronic phase structure. It is largest in the metallic regime, where abundant low-energy electron–hole excitations exist at the Fermi level. In the topological phase at an edge, friction is enhanced relative to a gapped bulk but remains below the metallic value because edge–LUMO hybridization produces an avoided crossing that splits the molecular projected density of states and depresses the local density of states precisely around μ. In the trivial phase and in the bulk of the topological phase away from edges, the electronic gap suppresses friction. These trends persist across adsorbate–substrate coupling strengths, with weaker coupling reducing the hybridization splitting and partially restoring the LDOS at μ.

These results suggest that low-dimensional topological substrates enhance adsorbate hybridization by exploiting localized midgap states at edges and domain walls for charge donation and promote adsorbate vibrational energy dissipation via enhanced electronic friction. Extensions to higher dimensions are promising: topological materials with localized boundary modes and appreciable boundary LDOS near μ (e.g., three-dimensional topological insulators or topological semimetal surfaces with robust surface states) are expected to further strengthen both electron donation and adsorbate friction, subject to the same resonance and overlap constraints discussed in this study. Finally, strategically patterning domain walls or stabilizing edge-rich morphologies offers a practical route to translate topological midgap physics into tunable knobs for adsorption energetics and vibrational relaxation in catalysis and surface chemistry.

Finally, our choice of a spinless, weakly correlated SSH model ensures that our modeling captures symmetry-protected midgap orbitals at edges and domain walls and their hybridization with an adsorbate. In more complex topological materials of interest for catalysis, a detailed treatment of spin degrees of freedom, strong spin–orbit coupling, and strong electron–electron correlations may reshape the low-energy spectrum, change the relevant symmetry class, and open additional relaxation channels. Extending the present framework to spinful tight-binding models (including SOC) and to beyond-mean-field-correlated descriptions (e.g., SSH–Hubbard-type models) is an important direction for quantifying the robustness of the trends identified here and for exploring regimes where electronic correlations qualitatively alter charge transfer and dissipation at molecule–surface interfaces.

## Supplementary Material



## Notes

We provide evidence of lack of size dependence of our results by reporting LUMO electronic occupation numbers and electronic friction versus SSH chain length *N* and discuss the Gaussian broadening σ used in the friction calculation, including its effect on the adsorbate-projected DOS in the trivial and topological phases (PDF)
