# Supplementary material for: Topological Advantage for Adsorbate Chemisorption on Conjugated Chains
Source: J Phys Chem Lett. 2026 Jan 27;17(6):1760–9. doi: 10.1021/acs.jpclett.5c03500 (PMC12908151; doi:10.1021/acs.jpclett.5c03500)
Supplement: Supplementary file 1 [file jz5c03500_si_001.pdf]

Supporting Information for Topological advantage for adsorbate chemisorption on conjugated chains

Luis Fernando Martinez-Gomez[1] and
Raphael F. Ribeiro[1]
[1]*Department of Chemistry, Emory University*
(Dated: December 26, 2025)

1. FINITE-SIZE CONVERGENCE

1.1. SSH chain length dependence

To assess finite-size effects, we recompute here (i) the adsorbate occupancy (electron donation) and (ii) the electronic friction for open SSH chains of several lengths, $N \in \{400, 600, 800, 1000\}$ unit cells, keeping all other parameters fixed. We parametrize the dimerization by $r := w/v$, for which $r < 1$ is the trivial insulator, $r = 1$ is the metallic point, and $r > 1$ is the topological phase (supporting edge modes for open boundaries). We consider two representative adsorbate placements: an edge placement x_E and a bulk placement x_B. When we write $\varepsilon_0(R, x_M)$, the label x_M indicates the chosen placement used to define the geometry-dependent adsorbate level energy.

Figure 1 shows the adsorbate occupancy as a function of r, comparing an edge placement (x_E) to a bulk placement (x_B). The edge occupancy exhibits only weak dependence on N once N is moderately large, consistent with the localized nature of edge-associated hybridization. In contrast, the bulk response is appreciable only in a narrow window near $r \simeq 1$, since the bulk LDOS at the chemical potential (here $\mu = 0$) is strongly suppressed in gapped phases ($r \neq 1$). The bulk panel is therefore shown on a zoomed r-range around the metallic point.

Figure 2 reports the friction γ as a function of the (placement-dependent) adsorbate level energy $\varepsilon_0(R, x_M)$ for the same set of chain lengths. Across all tested N, the metallic bulk placement yields a substantially larger friction than the topological edge placement, while gapped phases yield negligible friction. Importantly, the ordering and qualitative trends (metallic bulk $\gg$ topological edge $\gg$ gapped) are robust with respect to N.

1.2. Numerical regularization of the projected DOS for friction

For a finite chain the projected density of states entering the friction expression is a discrete sum of Dirac distributions at the single–particle eigenenergies $\{\varepsilon_n(R)\}$. For numerical stability we replace each δ–peak by a Gaussian of standard deviation σ,

$$\delta(\varepsilon - \varepsilon_n) \;\longrightarrow\; g_\sigma(\varepsilon - \varepsilon_n) := \frac{1}{\sqrt{2\pi}\,\sigma} \exp\left[-\frac{(\varepsilon - \varepsilon_n)^2}{2\sigma^2}\right], \tag{1}$$

which defines a smooth broadened spectral density $P(\varepsilon, R)$ (and the projected quantity $\langle \varepsilon_0 | P(\varepsilon, R) | \varepsilon_0 \rangle$ used below). The parameter σ must be small enough to resolve relevant spectral features (e.g., near the chemical potential) yet large enough to suppress spurious finite–size oscillations in energy integrals.

The friction integrand (see main text) is controlled by the overlap of the broadened (projected) DOS with the thermal window factor $-\partial_\varepsilon f_T(\varepsilon)$, where

$$f_T(\varepsilon) = \frac{1}{e^{\beta(\varepsilon-\mu)} + 1}, \qquad -\partial_\varepsilon f_T(\varepsilon) = \frac{\beta}{4\cosh^2\left(\frac{\beta(\varepsilon-\mu)}{2}\right)}. \tag{2}$$

This window is peaked at $\varepsilon = \mu$ and has full width at half maximum $\mathrm{FWHM}[-\partial_\varepsilon f_T] \approx 3.53\,k_BT$ (more precisely $4\,\mathrm{arccosh}(\sqrt{2})\,k_BT$). Throughout the main manuscript we set the *Gaussian* broadening to match this thermal smearing scale by equating FWHMs,

$$\mathrm{FWHM}[g_\sigma] = 2\sqrt{2\ln 2}\,\sigma \;\stackrel{!}{=}\; \mathrm{FWHM}[-\partial_\varepsilon f_T] \approx 3.53\,k_BT, \qquad \Rightarrow \quad \sigma_{\mathrm{th}} \approx \frac{3.53}{2\sqrt{2\ln 2}}\,k_BT \approx 1.50\,k_BT. \tag{3}$$

This choice (i) ties the numerical smoothing to a physically relevant energy scale and (ii) avoids introducing an artificial N–dependent broadening as the chain length increases.

While $\sigma_{\rm th}$ is the default choice used in the main text, we also test *different* broadenings to confirm that the qualitative friction trends are stable with respect to the numerical smoothing procedure. Figure 3 compares $\gamma(R)$ computed with two representative broadenings ($\sigma = 0.019$ and $\sigma = 0.038$), which differ from the thermal–matched value used in the manuscript, yet preserve the same qualitative ordering and physical interpretation.

In the metallic bulk ($r = 1$, x_B), decreasing σ sharpens the LDOS near μ, increasing the friction peak, whereas larger σ redistributes spectral weight away from μ and reduces γ. In the topological edge case ($r = 1.1$, x_E), $\gamma(R)$ is typically less sensitive to σ because the bulk gap eliminates low–energy bulk excitations and the relevant low–energy spectral weight is dominated by the edge–adsorbate hybridization.

When the adsorbate level is near resonance with the edge mode, the dominant effect of the molecule–boundary coupling $T_{1,A}$ is an avoided crossing between the edge state and the adsorbate state. To leading order this produces a PDOS doublet on the adsorbate with splitting

$$\Delta E \;\simeq\; 2\,|V|, \qquad V \sim T_{1,A}\,\alpha_1^L, \tag{4}$$

where α_1^L is the edge–mode amplitude on the boundary site to which the adsorbate couples (so V is the effective edge–adsorbate hybridization matrix element). When ΔE is large, the two PDOS peaks flank μ, reducing their overlap with $-\partial_\varepsilon f_T$ and suppressing $\gamma(R)$. Reducing $T_{1,A}$ decreases ΔE, which can increase the overlap and thereby increase $\gamma(R)$ over an intermediate range of couplings. *In the asymptotically weak–coupling limit* $T_{1,A} \to 0$, however, the adsorbate decouples from the chain and $\gamma(R)$ must ultimately vanish.

Figure 4 provides a direct visualization of the mentioned overlap mechanism by plotting, on a common energy axis, the adsorbate–projected spectral weight and the thermal window factor $-\partial_\varepsilon f_T(\varepsilon)$. For each color, the solid and dashed curves should be compared: solid lines report the squared projected density of states of the adsorbate, $\left|\langle\varepsilon_0|\,P(\varepsilon_\lambda)\,|\varepsilon_0\rangle\right|^2$, whereas dashed lines show $-\partial_\varepsilon f_T(\varepsilon)$. Panel 4(a) shows the topological phase for an edge-bound adsorbate at resonance, $\varepsilon_0(R, x_E) = 0$, for several adsorbate–chain couplings. As the coupling increases, the midgap peak in the projected spectrum splits and shifts away from $\epsilon_\lambda = 0$, thereby decreasing its overlap with the thermal window and reducing the friction. Panel 4(b) shows the dependence on the dimerization ratio $r = w/v$ at resonance for metallic bulk ($r = 1.0$) and topological edge ($r = 1.1$) placements. In this case, the overlap is maximized when the projected spectral weight is centered near the chemical potential μ, reflecting that the electronic friction of the adsorbate is maximized in the metallic phase.

Across the tested σ values (including the thermal–matched choice used in the main manuscript and the distinct values shown in Fig. 3), our qualitative conclusions are unchanged: (i) metallic bulk friction exceeds topological–edge friction due to larger low–energy spectral weight near μ; (ii) gapped regions yield negligible friction; and (iii) tuning the molecule–edge coupling modifies the hybridization splitting and hence the spectral overlap with the thermal window, without changing the qualitative ordering of regimes.

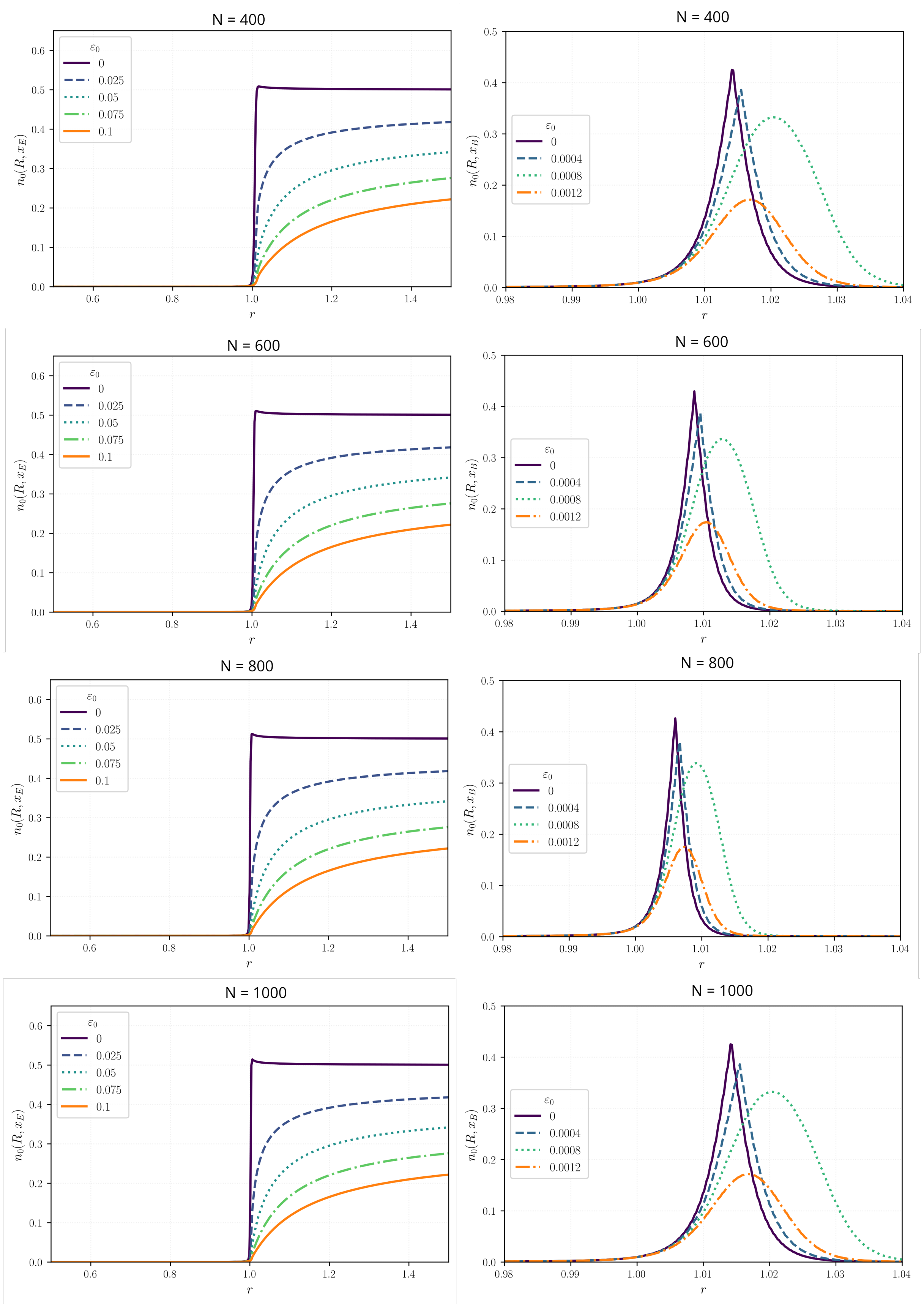

FIG. 1. Dependence of the adsorbate occupancy (electron donation) on the dimerization ratio $r = w/v$ for different SSH chain lengths N. Left: edge placement x_E. Right: bulk placement x_B (panel shown on a zoomed r-range around the metallic point). Parameters: $v = 10$, $\mu = 0$. (The values of ε_0 are indicated in the legend.)

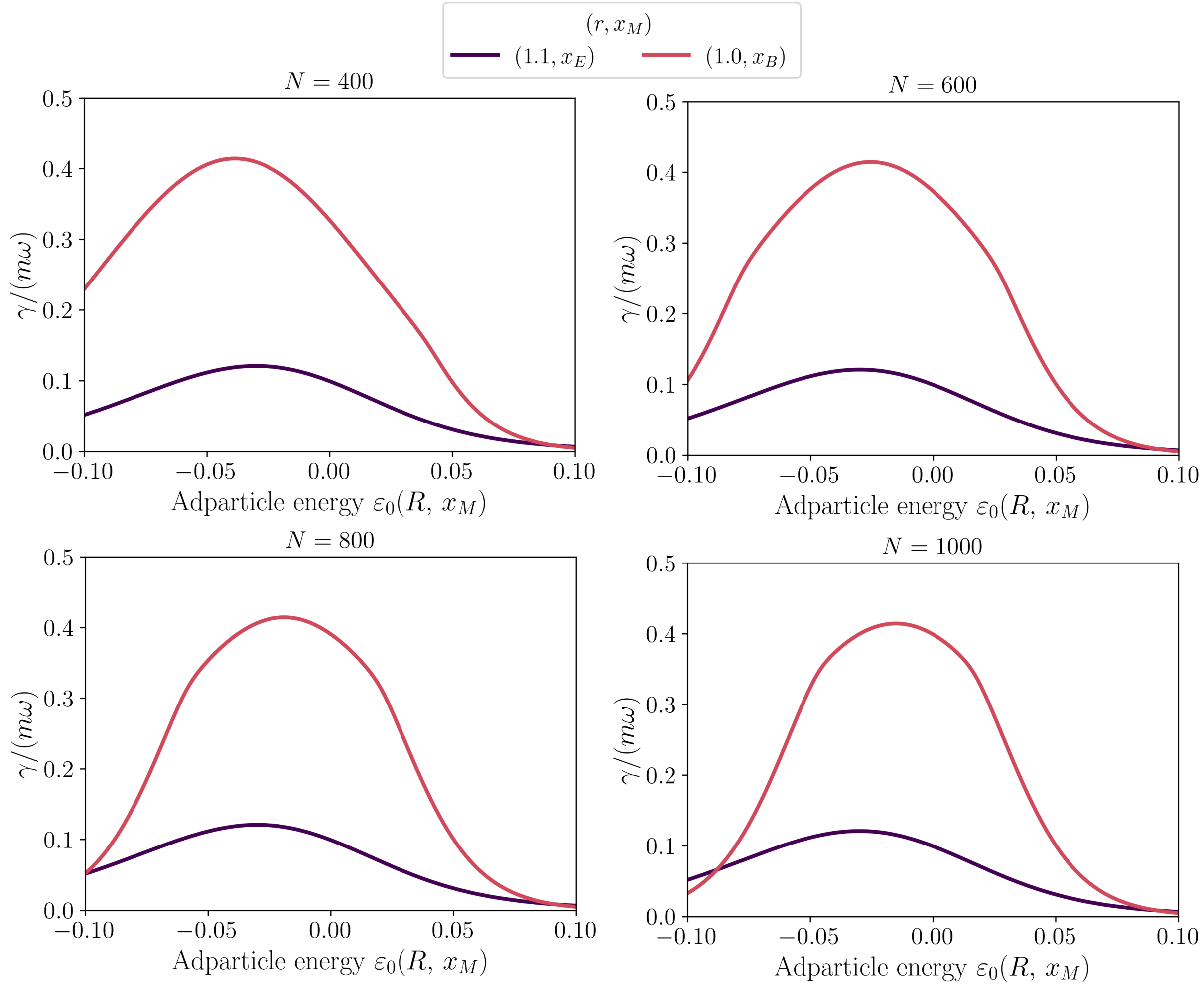

FIG. 2. Dependence of the electronic friction γ on the adsorbate level energy $\varepsilon_0(R, x_M)$ for different SSH chain lengths N. Metallic bulk: $r = 1$, x_B (red). Topological edge: $r = 1.1$, x_E (dark purple). Parameters: $v = 10$, $\sigma = 0.0225$, $\varepsilon_d = 0.15$, $g = 0.02$, $\beta = 1/0.015$, and $T_{1,A} = T_{N/2,A} = 0.1$ with $T_{N/2-1,B} = T_{N/2,B} = T_{N/2,A}/3$.

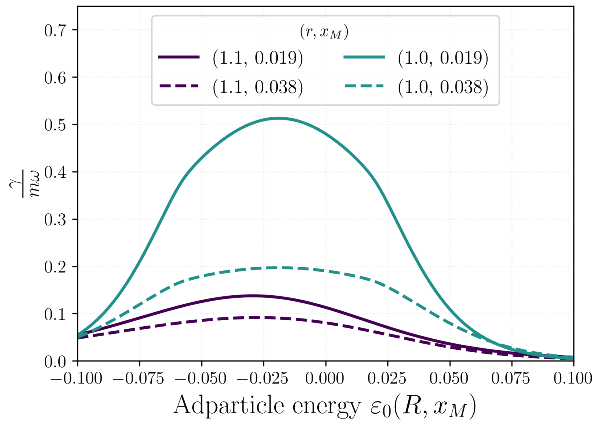

FIG. 3. Dependence of $\gamma(R)$ on the Gaussian broadening σ in the topological edge case ($r = 1.1$, x_E) and in the metallic bulk case ($r = 1$, x_B). Two values are shown: $\sigma = 0.019$ and $\sigma = 0.038$. Other parameters: $v = 10$, $g = 0.02$, $\beta = 1/0.015$, $N = 800$, $T_{1,A} = T_{N/2,A} = 0.1$, and $T_{N/2-1,B} = T_{N/2,B} = T_{N/2,A}/3$.

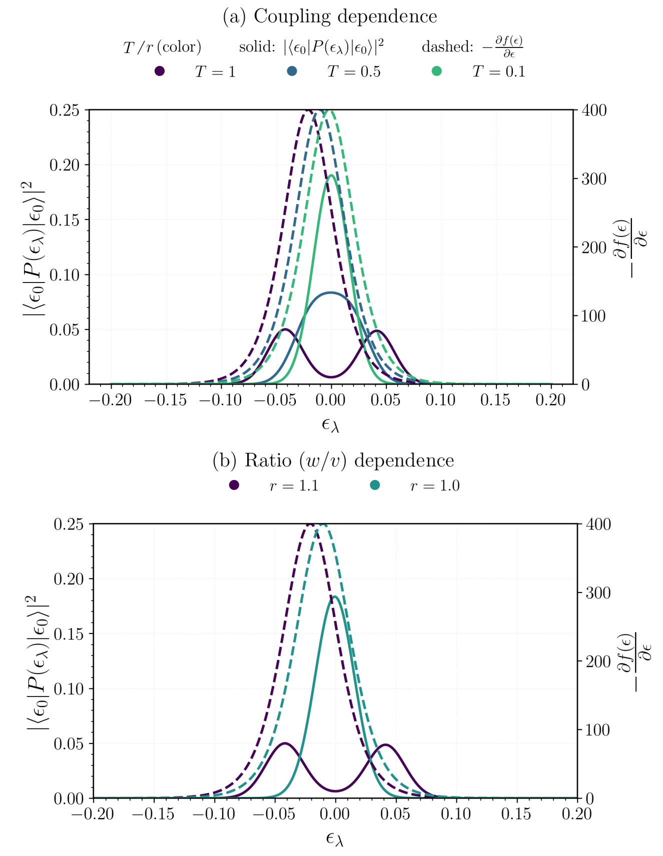

FIG. 4. Overlap between the adsorbate–projected spectrum and $-\partial_\varepsilon f_T$. Solid lines (left axis) show the squared projected density of states PDOS of the adsorbate $\left|\langle\varepsilon_0|\, P(\varepsilon_\lambda)\,|\varepsilon_0\rangle\right|^2$, while dashed curves (right axis) show the derivative of the Fermi–Dirac distribution, $-\partial_\varepsilon f_T(\varepsilon)$. (a) Dependence on the adsorbate–SSH coupling in the topological phase for an edge-bound adsorbate at resonance, $\varepsilon_0(R, x_E) = 0$. Increasing the coupling splits the midgap peak and reduces its overlap with the thermal window. (b) Dependence on the dimerization ratio $r = w/v$ for bulk ($r = 1.0$) and edge ($r = 1.1$) placements at resonance. Here the overlap is maximized when the projected spectral weight is centered near the chemical potential μ. Parameters: $\varepsilon_0(R, x_M) = 0$, $v = 10$, $g = 0.02$, $\sigma = 0.0225$, $\beta = 1/0.015$, $N = 800$, $T_{1,A} = T_{N/2,A} = 0.1$, and $T_{N/2-1,B} = T_{N/2,B} = T_{N/2,A}/3$.
